# Screening of Inhibitory Effects of Polyphenols on Akt-Phosphorylation in Endothelial Cells and Determination of Structure-Activity Features

**DOI:** 10.3390/biom9060219

**Published:** 2019-06-05

**Authors:** Stoyan Dirimanov, Petra Högger

**Affiliations:** Institut für Pharmazie und Lebensmittelchemie, Universität Würzburg, Am Hubland, 97074 Würzburg, Germany; stoyan.dirimanov@uni-wuerzburg.de

**Keywords:** Akt/PKB, endothelium, diabetes, polyphenols, in vitro, structure-activity relationships, screening

## Abstract

Polyphenols exert beneficial effects in type 2 diabetes mellitus (T2DM). However, their mechanism of action remains largely unknown. Endothelial Akt-kinase plays a key role in the pathogenesis of cardiovascular complications in T2DM and therefore the modulation of its activity is of interest. This work aimed to characterize effects of structurally different polyphenols on Akt-phosphorylation (pAkt) in endothelial cells (Ea.hy926) and to describe structure-activity features. A comprehensive screening via ELISA quantified the effects of 44 polyphenols (10 µM) on pAkt Ser473. The most pronounced inhibitors were luteolin (44 ± 18%), quercetin (36 ± 8%), urolithin A (35 ± 12%), apigenin, fisetin, and resveratrol; (*p* < 0.01). The results were confirmed by Western blotting and complemented with corresponding experiments in HUVEC cells. A strong positive and statistically significant correlation between the mean inhibitory effects of the tested polyphenols on both Akt-residues Ser473 and Thr308 (r = 0.9478, *p* = 0.0003) was determined by immunoblotting. Interestingly, the structural characteristics favoring pAkt inhibition partially differed from structural features enhancing the compounds’ antioxidant activity. The present study is the first to quantitatively compare the influence of polyphenols from nine different structural subclasses on pAkt in endothelial cells. These effects might be advantageous in certain T2DM-complications involving over-activation of the Akt-pathway. The suggested molecular mode of action of polyphenols involving Akt-inhibition contributes to understanding their effects on the cellular level.

## 1. Introduction

**Figure 1:**
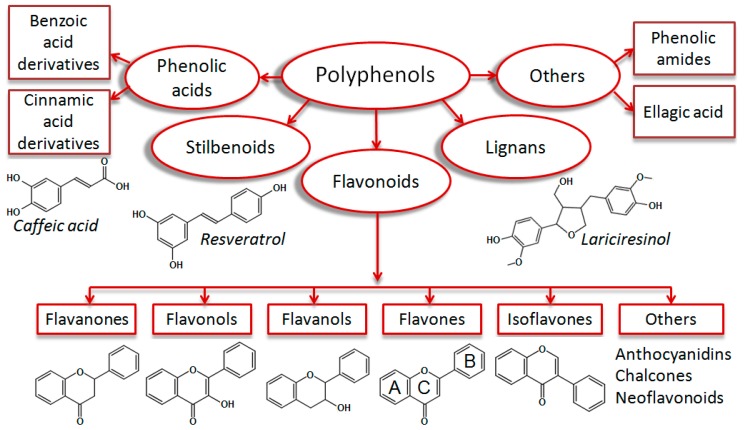
Classification of monomeric polyphenolic compounds according to Zhang et al. 2016 [2], with some modifications. Polyphenols are plant-derived compounds of four main structural subclasses: Phenolic acids, stilbenoids, flavonoids, and lignans. Phenolic acids are derivatives of benzoic or cinnamic acids (i.e., caffeic acid). Stilbenoids are hydroxylated stilbene derivatives (i.e., resveratrol). Lignans are diphenolic compounds (i.e., lariciresinol). Flavonoids are phenylchromone derivatives, which are subgrouped into flavanones, flavonols, flavanols, flavones, isoflavones, and others (anthocyanidins, chalcones, and neoflavonoids). Some authors classify phenolic amides (i.e., capsaicin) and ellagic acid derivatives [3] as additional polyphenol groups.

Polyphenols are plant secondary metabolites which are ubiquitously present in human food, such as fruits, vegetables, nuts, spices and beverages [1]. These structural diverse phytochemicals are important micronutrients in the human diet. According to their scaffolds, polyphenols are typically classified as flavonoids, stilbenoids, lignans, and phenolic acids [2] (Figure 1).

The group of flavonoids (phenylchromones) comprises the highest number of representatives (around 4000), which are further subdivided into flavones, flavonols, flavanones, flavanols, isoflavonoids, anthocyanidins, neoflavonoids and chalcones. A multitude of substitution patterns in the A and B rings yields various derivatives within each subclass of flavonoids. Typical substituents are hydroxyl and methoxy groups, *O*-glycosides, sulfates, and glucuronides [4].

After ingestion of high molecular weight polyphenols, low molecular weight metabolites can be produced by the intestinal microflora [5]. Thereby, compounds might undergo bio-activation and exhibit stronger effects than their direct metabolic precursor molecule [6]. This has been shown for the procyanidin and ellagitannin metabolites δ-(3,4-dihydroxy-phenyl)-γ-valerolactone and urolithins, respectively [7,8].

Various in vitro [9], in vivo [10,11,12], and clinical studies [13,14,15] suggested that polyphenols might have beneficial effects regarding diabetes-induced complications of the cardiovascular system. In certain cases, it was described that these effects are mediated through Akt-kinase [10,11].

**Figure 2:**
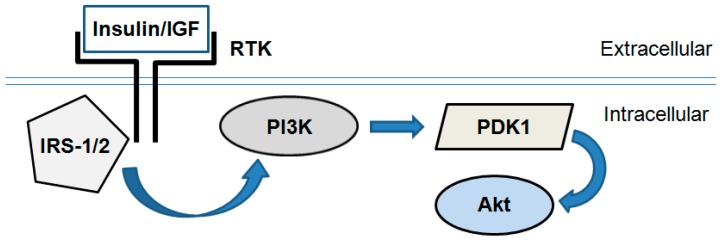
Simplified scheme of the PI3K/Akt signaling pathway. Insulin or insulin-like growth factor-1 (IGF) binds and activates receptor tyrosine kinases (RTK; i.e., insulin receptor). In turn they activate an intracellular signal transduction cascade consisting of several enzymes: Insulin receptor substrate-1/2 (IRS-1/2), phosphoinositide-3-kinase (PI3K), phosphoinositide-dependent kinase 1 (PDK1) and Akt/PKB.

PI3K/Akt is a key signaling pathway responsible for fundamental and mainly anabolic cellular processes, such as protein synthesis, glucose uptake and metabolism, proliferation and cell survival [16]. This pathway mediates insulin metabolic effects on the cellular level and comprises a cascade of signal molecules such as insulin receptor, insulin receptor substrates, phosphoinositide-3-kinase (PI3K), phosphoinositide-dependent kinase 1 (PDK1), and Akt (also known as Protein Kinase B, PKB, Figure 2).

In general, insulin- or insulin-like growth factor (IGF)-induced Akt activation is governed by PI3K, which is directly phosphorylated and activated by insulin receptor substrate-1/2 (IRS-1/2). In turn, PI3K produces the lipid second messenger phosphatidylinositol-(3,4,5)-trisphosphate (PIP3). It activates PDK1 and interacts with the pleckstrin homology domain of Akt resulting in its recruitment to the plasma membrane. PDK1 phosphorylates Akt at a threonine (Thr308) site and thus initiates its activation [17].

Presently three Akt isoforms, Akt1/PKBα, Akt2/PKBβ, and Akt3/PKBγ, are known. They are structurally similar, but functionally different [18]. Insulin has differential effects on the subcellular distribution of Akt1 and Akt2, which indicates distinct physiological functions for the two isoforms. Akt2 showed a more pronounced accumulation in the membrane compartment compared to Akt1. This correlates with the specific role shown for Akt2 regarding the regulation of GLUT-4 (glucose transporter type 4) trafficking and insulin-mediated glucose transport [19].

Akt-kinase contributes in mediating intracellular effects of insulin and is therefore involved in the pathophysiology of diabetes and its vascular complications [20]. There is evidence that the Akt-signaling pathway might be altered in T2DM and this might contribute to insulin resistance [21]. In this pathological state an imbalance characterized by a prevalence of the mitogen-activated protein kinase (MAPK) signaling over PI3K/Akt pathway has been described [22].

In addition, Akt can affect further processes associated with T2DM and its long-term consequences. These include glucose transport, glycogen synthesis [23], apoptosis [24], endothelial dysfunction [22], and angiogenesis [25]. Therefore, modulators of Akt-phosphorylation and respectively activity are extensively investigated as pharmacological tools [26].

Akt is activated through phosphorylation at various sites, the most important are the amino acids threonine 308 (Thr308) and serine 473 (Ser473). The direct upstream kinase responsible for Thr308 phosphorylation is PDK1, while Ser473 phosphorylation is primarily mediated through the mTORC2 (mechanistic target of rapamycin complex 2) pathway [17].

In addition to its pro-survival effects, Akt is involved in angiogenesis, vasorelaxation and vascular remodeling processes [26]. The Akt1 isoform is predominantly expressed in the endothelium. It has been reported that activation of Akt-kinase in endothelial cells (EC) contributes to the cardio-metabolic homeostasis by subsequent activation of eNOS and release of NO, and has protective properties regarding long-term vascular complications of T2DM [27].

Pathological neovascularization has been observed in patients with metabolic syndrome, indicating progression of microvascular complications such as nephropathy and retinopathy [28,29]. Excessive neo-angiogenesis was also described to contribute to atherosclerotic plaque formation and vulnerability [30]. Since Akt-kinase participates in angiogenesis [25,31], it is conceivable that over-activation of Akt plays a role in the pathogenesis of diabetic vascular complications. Indeed, a study demonstrated that increased glucose levels contributed to neovascularization in diabetic retinopathy in vivo, which was mediated through elevated basal Akt-phosphorylation and inhibition of the latter prevented the process [32]. The use of anti-angiogenic agents in the treatment of these complications has been proposed [29]. Since certain polyphenols have been described to exhibit anti-angiogenic properties [33] this could contribute to their beneficial role in diabetes.

The potential relationship between health promoting properties of polyphenolic compounds and their capability to modulate insulin signal transduction demanded a comprehensive study regarding the effects of individual polyphenols on the phosphorylation of Akt-kinase (pAkt). The aim was to identify subclasses and representatives of polyphenols that modulate this signaling pathway and thus might be effective in the prevention and management of T2DM late complications. Therefore, quantitative effects of 44 polyphenolic compounds and metabolites on the phosphorylation of Akt (Ser473) in endothelial cells in vitro were determined via ELISA and confirmed by Western blot analysis.

## 2. Materials and Methods

### 2.1. Chemicals and Reagents

Polyphenols (listed with their purity) purchased from Tokyo Chemical Industry (TCI) Co., Ltd., Tokyo, Japan, included resveratrol (>98%), pinostilbene (>97%), pterostilbene (>98%), 3,4′,5-trimethoxy-trans-stilbene (>96%), piceatannol (>98%), oxyresveratrol (>95%), naringenin (>93%), apigenin (>98%), taxifolin (≥85%), genistein (>96%), 3-hydroxyflavone (>98%), 3-methoxyflavone (>98%), 7-methoxyflavone (>98%), 3,4′-dihydroxy–flavone (>97%), 3-hydroxy-4′-methoxyflavone (>98%), kaempferol (>97%), myricetin (>97%), chrysin (>97%), fisetin (>96%), baicalein (>98%), 6-hydroxyflavone (>98%), 6-methoxyflavone (>98%), 7,8-dihydroxy-flavone (>98%), (−)-epigallocatechin gallate (>98%), flavanone (>98%). Urolithin A, C and D were obtained from Newchem Technologies Ltd., Durham, UK. Urolithin B (≥95%), flavone (≥99%), chlorogenic acid (>95%), morin (>85%), quercetin (>98%), caffeic acid (≥98%), (+)-catechin (>99%), ellagic acid (>95%), were from Sigma-Aldrich, St. Louis, MO, USA. Luteolin (≥98%), trans-ferulic acid (>99%), (−)-epicatechin (≥98%), baicalin (>95%), wogonoside (≥98%), (−)-gallocatechin gallate (≥98%), (−)-epigalocatechin (≥98%) were from Aladdin, Shanghai, China. Vitexin (≥98%) was a product of TAUTO^®^, Shanghai, China.

Catechin metabolites M1 [δ-(3,4-dihydroxyphenyl)-γ-valerolactone] and M2 [δ-(3-methoxy -4-hydroxyphenyl)-γ-valerolactone] were synthesized by M. Rappold and kindly provided for use.

Stock solutions (10^−2^ M) of the polyphenols in DMSO were prepared and stored at –80 °C or directly used for cell culture experiments.

### 2.2. Cell Culture

The immortalized human endothelial cell line Ea.hy926 was generously provided for use from Dr. C.J. Edgell (University of North Carolina, Chapel Hill, NC, USA) [34]. Ea.hy926 cells were used between passages 6 and 30. Cells were cultured according to a standard protocol [35] in Dulbecco’s modified essential medium, high glucose (HG DMEM, 4500 mg/L glucose; Sigma-Aldrich, St. Louis, MO, USA) without phenol red because this compound has been described to possess estrogen-like properties [36]. Medium was supplemented with 10% heat inactivated fetal bovine serum (FBS; Batch No. 1107A; Biochrom AG, Berlin, Germany), 3.7 g/L NaHCO_3_, 2 mM l-glutamine, 1 mM non-essential amino acids (NEA), 1 mM sodium pyruvate, and a mixture of 100 U/mL penicillin with 100 μg/mL streptomycin (1% Pen/Strep) at 37 °C in 5% CO_2_ atmosphere. Briefly, cells were seeded into 75 cm^2^ flasks (Sarstedt AG & Co., Nümbrecht, Germany) at a density of 2000–2500 cells/cm^2^. The medium was changed every second day. When cells reached 90% confluence they were passaged. For this purpose, they were rinsed three times with warm Dulbecco’s phosphate buffered saline (PBS) and treated with trypsin/EDTA solution 1× (trypsin 0.05%/EDTA 0.02%; Sigma-Aldrich, St. Louis, MO, USA). Detached cells were resuspended in a fresh warm medium. Cell suspension was either seeded into flasks for further cultivation or into 6-well plates at a density of 0.3 × 10^6^ cells per well for experiments.

Primary endothelial cells HUVEC were cultivated according to the protocol described above with some deviations. Instead of DMEM, M 199 (M3769) supplemented with 4500 mg/L glucose (final concentration), 2.2 g/L NaHCO_3_, 20% heat inactivated FBS, 2 mM l-glutamine, 1% low serum growth supplement (LSGS), and 1% Pen/Strep was used. All flasks and wells used for HUVECs were coated with a sterile 1% gelatin solution. After trypsinization cells were mixed with growth medium and pelleted by centrifugation (7 min, 25 °C, 1200 g) in order to remove the proteolytic enzyme. Then the cells were resuspended in a fresh warm M 199 and passaged or seeded for an experiment as indicated. Trypan blue staining was routinely performed to determine the vitality of cells.

Cells were grown in a 3 mL medium/well until they reached confluence. Afterwards, they were incubated with polyphenolic compounds at a concentration of 10 µM, which was considered as a physiologically relevant concentration [37]. Due to the chemical instability of some polyphenols [38] the incubation time was limited to 5 min. Individual polyphenols were added to cells in standard DMEM with 10% FBS (batch 1107A) to determine their potential to antagonize the growth factor-induced Akt-phosphorylation and to mimic physiological conditions. As a positive control cells were incubated in a serum-free medium overnight. Absence of growth factors strongly decreased Akt-phosphorylation [39].

### 2.3. Cell Lysis and Sample Preparation

After treatments cells were rinsed twice with ice-cold PBS and 300 µL precooled lysis buffer (CelLytic™ M, Sigma-Aldrich, St. Louis, MO, USA, or lysis buffer provided with the ELISA kit), supplemented with phosphatase and protease inhibitors (PhosSTOP™, Roche Diagnostics GmbH, Mannheim, Germany; Protease Inhibitor cocktail for mammalian cells and tissue extracts, P8340 from Sigma-Aldrich, St. Louis, MO, USA), were added to each well. Cellular proteins were extracted at 4 °C on a shaker for 15 min. Cell residues were scraped off the wells and the lysates were transferred to 1.5 mL precooled plastic tubes and centrifuged at 4 °C for 10 min at 18,000 g. The supernatants were transferred into fresh tubes, vortexed, and briefly sonicated. If needed, protein concentration in extracts was determined via BCA assay (Pierce^®^ BCA Protein Assay Kit) according to the protocol provided by the manufacturer (Thermo Fisher Scientific, Waltham, MA, USA).

### 2.4. ELISA

For the screening of pAkt, RayBio^®^ Human/Mause/Rat Phospho-Akt (S473) and Total Akt ELISA Kit (Raybiotech, Inc., Peachtree Corners, GA, USA) was used according to the manufacturer’s protocol with some deviations. Lysates were diluted 1:3 with (1×) assay diluent and were added to assay wells and incubated overnight at 4 °C. In each experiment, the lysate from DMSO-treated cells (corresponding to the 100% control) and its 1:1 dilution (50% control) were used as reference standards. The antibody detecting pAkt (Ser473) was diluted 1:55 in (1×) assay diluent as suggested by manufacturer, while pan (total) Akt antibody was diluted 1:220 in order to avoid readouts outside the linear range of the assay. Based on optical density readings after blank subtraction, values (in %) for pAkt (Ser473) and pan Akt were calculated using the reference standards. Then data for pAkt (Ser473) were normalized with reference to pan Akt.

### 2.5. Western Blot 

For Western blot, samples were mixed with Laemmli buffer (4×) and DTT (dithiothreitol). After incubation for 7 min at 70 °C under shaking (1000 rpm) they were vortexed, shortly spun and either directly analyzed or stored at –20 °C. Western blot was performed with phosphospecific antibodies against pAkt Ser473 (dilution: 1:1000) and pAkt Thr308 (1:800, all antibodies from Cell Signaling Technology, Inc., Danvers, MA, USA). Pan Akt (1:2000) was used as a loading control. 

Proteins were separated by SDS PAGE (Mini-PROTEAN Tetra^®^, Gel-Electrophoresis Equipment, Bio-Rad Laboratories, Inc., Grand Junction, CO, USA) using 5% stacking and 10% resolving polyacrylamide gels (Rotiphorese Gel 30 (37.5:1) from Carl Roth GmbH + Co, Karlsruhe, Germany). Gels were loaded with equal protein concentrations (20–30 µg/lane). Proteins were subsequently transferred onto nitrocellulose membranes using wet blotting (Mini-Trans Blot^®^ cell, Bio-Rad Laboratories, Inc., Grand Junction, CO, USA). The process took one hour and was performed at 4 °C and 375 mA/100 V.

Membranes were blocked for one hour at room temperature using 5% BSA in TBST (Tris-buffered saline, 0.05% Tween 20) in case of phospho-Akt (Thr308 and Ser473) and 5% low-fat dry milk powder (J.M. Gabler–Saliter Milchwerk GmbH & Co. KG, Obergünzburg, Germany) in TBST for pan Akt membranes. After a brief wash with TBST, primary rabbit antibodies in 5% BSA-TBST were applied and incubated at 4 °C overnight on a shaker. To remove the unbound primary antibodies, membranes were washed four times for 10 min with TBST. A secondary, HRP-linked anti-rabbit antibody (dilution: 1:10000) was applied for two hours at room temperature (or at 4 °C overnight, alternatively). To reduce signal/noise ratio, membranes were again washed four times for 10 min with TBST. A chemiluminescent detection (Clarity™ Western ECL substrate; Bio-Rad Laboratories, Inc., Grand Junction, CO, USA) using the FluorChem FC2 Doku imaging system (Alpha Innotec GmbH, Kasendorf, Germany) was performed. The images were quantified densitometrically using of ImageJ [40]. After detection of pAkt, membranes were stripped/reprobed for detection of total (pan) Akt. For this purpose, a standard stripping buffer (200 mM glycine, 0.1% (*w*/*v*) SDS, 1.0% (*v*/*v*) Tween 20 in Millipore water, pH = 2.2) was used.

### 2.6. Statistical Analysis 

Mean values, standard deviations (S.D.), medians, and mean deviation (Mean Dev.) were calculated with Microsoft Excel^®^, Version 2010. For multiple comparisons, analysis of variance (one-way ANOVA) was utilized. The statistical significance of the differences between two samples was examined using a two-tailed paired Student’s *t*-test. The significance level α was set to 0.05. The tests were performed with the Real Statistics Add-in^©^ (2013–2016, Charles Zaiontz) of Excel [41]. Alternatively, the freely available online web calculator Astatsa (2016, Navendu Vasavada) was used for post-hoc Tukey HSD (honestly significant difference) test after multiple group comparisons [42].

## 3. Results

### 3.1. Screening for Short-Term Effects of Polyphenols on pAkt

The comprehensive screening regarding short-term effects of polyphenols on the phosphorylation of Akt at Ser473 included 44 compounds with different structures and certain microbiota-generated metabolites (Table 1).

Overall, 26 compounds revealed some inhibitory potential. Among them, 11 substances showed pronounced (> 20%), 10 compounds lower, but still distinguishable (between 10% and 20%), and five compounds weak (less than 10%) inhibitory activity. The other 18 polyphenols did not inhibit pAkt (Supplemental Materials; Table S1). The inhibitory activity was expressed as percent compared to vehicle-treated negative controls ± standard deviation (100%–residual phosphorylation).

**Figure 3:**
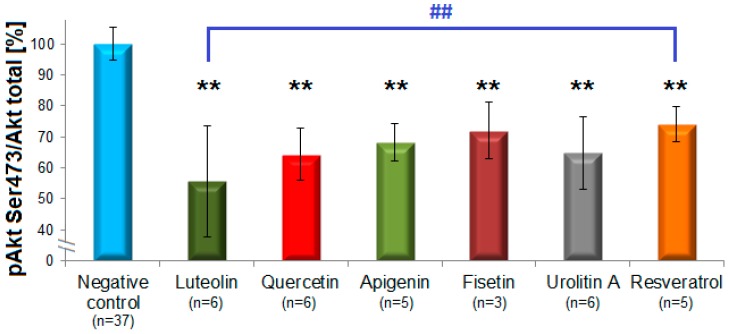
Polyphenols from different subclasses showing more pronounced pAkt-inhibition. The columns represent means of the residual phosphorylation (in %) with standard deviations. Statistically significant inhibitions were observed for the flavones luteolin and apigenin, the flavonols fisetin and quercetin, the dibenzo-α-pyrone urolithin A and the stilbenoid resveratrol (n = 3–6), ** *p* < 0.01. The difference between the effects of luteolin and resveratrol was statistically significant as well, ^##^ *p* < 0.01 (one-way ANOVA with Tukey HSD test).

Flavones and flavon-3-ols caused the clearest inhibition of Akt-phosphorylation (Figure 3). The most active compound was luteolin (44 ± 18%, n = 6), followed by quercetin (36 ± 8%, n = 6), apigenin (32 ± 6%, n = 5), fisetin (28 ± 9%, n = 3), 6-hydroxyflavone (27 ± 8%, n = 2), 6-methoxyflavone (25 ± 10%, n = 2), and flavone (22 ± 3%, n = 2). The subclass of flavanols possessed no inhibitory effects with the exception of (–)-gallocatechin gallate (12%). The representatives of flavanones (naringenin), isoflavones (genistein), and flavanonols (taxifolin) showed no considerable inhibition and were not further investigated. Among the group of stilbenoids, resveratrol (26 ± 5.6%, n = 5) and pinostilbene (19 ± 14, n = 2) were active, unlike pterostilbene, 3,4′,5-trimethoxy-trans-stilbene and piceatannol which did not inhibit pAkt. All three structural analogues from the subclass of phenolic acids (caffeic, ferulic and chlorogenic acids) showed no inhibitory effects. Notably, in the group of ellagic acid metabolites only urolithin A (35 ± 12%, n = 6), but not urolithin B, C or D inhibited pAkt.

(+)-Catechin, (−)-epicatechin, ferulic acid, M2, naringenin and all investigated glycosides (vitexin, wogonoside, baicalin) exhibited a non-statistically significant tendency to slightly augment pAkt Ser473.

Statistical significant effects of luteolin, urolithin A, apigenin, quercetin, fisetin, and resveratrol (*p* = 0.001) were determined by one-way ANOVA with the Tukey HSD test [42]. The statistical analysis also revealed a statistically significant difference between the effects of luteolin and resveratrol (*p* = 0.008; Figure 3).

### 3.2. Structure-Activity Relationship: Key Features

**Figure 4:**
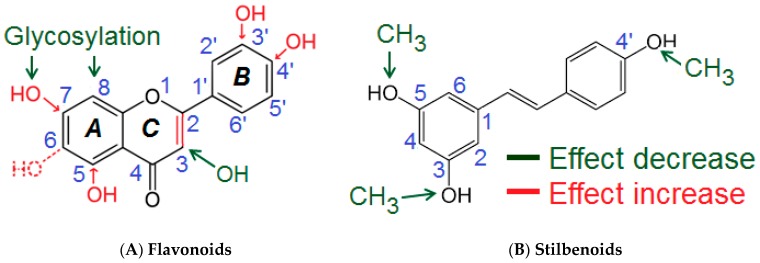
Proposed semi-quantitative structure-activity relationships for flavones/flavon-3-ols (**A**) and stilbenoids (**B**). Structural features decreasing the inhibition of Akt-phosphorylation are marked in green color. Structural characteristics increasing the inhibitory potential of polyphenolic compounds are shown in red. For more details please refer to the text and Table 2.

Based on the results of the comprehensive screening, a semi-quantitative assumption for the structural determinants influencing the polyphenols’ inhibitory activities on Akt phosphorylation at Ser473 was developed (Figure 4, Table 2).

Several molecular sites of the polyphenols appeared to be important for the inhibition of Akt-phosphorylation. The presence of a double bond C2=C3 (ring C) appeared to be essential for the inhibitory activity of flavones and flavon-3-ols. This was concluded from the comparison of the inhibitory effects caused by quercetin (36 ± 8%) compared to taxifolin (9%), and apigenin (32 ± 6%) compared to naringenin (−7%). Hydroxylation at C3 in the C-ring reduced the inhibitory potential of flavones/flavon-3-ols. Evidence for that were the differences between the effects of luteolin (44 ± 18%) compared to quercetin (36 ± 8%), and apigenin (32 ± 6%) compared to kaempferol (17 ± 12%). Methylation of the hydroxyl group (C3) partially prevented the decrease of the inhibitory activity as seen by comparing the effects of 3-methoxyflavone (13%) compared to 3-hydroxyflavone (6 ± 4%). Compounds featuring a single hydroxylation or methylation at C6-position in the A-Ring also possessed distinguishable inhibitory activities (>20%: 6-hydroxyflavone and 6-methoxyflavone). On the other hand, the most active flavones (luteolin, apigenin) were hydroxylated at the C5- and C7- position of the Ring A, which suggested that these structural features were important for the inhibitory potential. Since the Ring B of the most active flavones/flavon-3-ols (e.g., luteolin, fisetin, apigenin, quercetin) was hydroxylated, the OH groups (Ring B) obviously contributed to the inhibitory effects. The presence of a meta- and a para-OH groups appeared to be optimal for the activity as seen for luteolin (*m*, *p*-OH) > apigenin (*p*-OH) > chrysin (Ø OH); 3,4′-dihydroxyflavone (*p*-OH) > 3-hydroxyflavone (Ø OH). The investigated glycosides showed no effects on Akt-phosphorylation. This modification abolished the inhibitory potential as seen when comparing apigenin (32 ± 6%) compared to vitexin (−8.5%), baicalein (18%) compared to baicalin (−11%) and wogonoside (−5%).

For the subclass of stilbenoids, the presence of the three free OH groups in 3-, 4′-, and 5-positions appeared optimal for the inhibitory effects. Methylation of these groups reduced or even to completely eliminated pAkt inhibition (resveratrol (26 ± 5.6%) > pinostilbene (19 ± 14%) > pterostilbene (−6 ± 1.6%) ≈ 3,4′,5-trimethoxy-trans-stilbene (0.9 ± 10%)).

In the subclass of urolithins clear differences in their inhibitory effects were observed. As seen with urolithin A, two OH-groups at the C3 and C8 positions and lack of further substituents were important for the activity. Thus, only minor changes, such as an addition or elimination of a hydroxyl group were responsible for a remarkable change in the inhibition. Similar observation was valid for flavonols: Active quercetin and slightly active morin – analogs, differing only by the position of one phenolic OH-group in the B-ring.

To determine whether structural determinants responsible for the observed inhibitory effects on pAkt in the present study matched published structural features enhancing the polyphenols’ antioxidant properties [43], both activities were compared (Table 3; more details are described in the discussion section).

### 3.3. Possible Activation through Bio-Transformation

**Figure 5:**
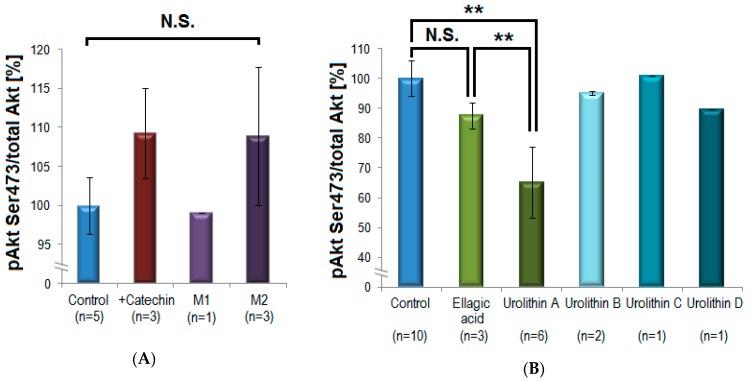
Investigation of a potential bio-activation of polyphenols by intestinal bacteria. (**A**) (+)-Catechin was compared with its microbiota-generated metabolites M1 and M2. (+)-Catechin and M2 caused non-significant (N.S.) slight increase in Akt-phosphorylation, M1 showed no activity. (**B**) Ellagic acid did not significantly influence the phosphorylation of Akt. In contrast, its microbial metabolite urolithin A induced a pronounced and statistically significant inhibition of Akt-phosphorylation compared to control (** *p* = 0.001, mean ± standard deviation) and compared to ellagic acid (** *p* = 0.005, one-way ANOVA/Tukey post-hoc test). Other urolithins showed only minor inhibitory effects (n = 3–6 for (+)-catechin, M2, ellagic acid, and urolithin A, n = 1–2 for other compounds).

The direct precursor compounds (+)-catechin and ellagic acid were compared with their corresponding intestinal microbiota-generated metabolites regarding their in vitro inhibitory potential on pAkt Ser473. (+)-Catechin caused a slight statistically non-significant increase of Akt-phosphorylation with 9 ± 6% (n = 3; mean inhibition ± S.D.), while M1 (δ-(3,4-dihydroxyphenyl)-γ-valerolactone) exhibited no influence on pAkt (n = 1), and the methylated M2 (δ-(3-methoxy-4-hydroxyphenyl)-γ-valerolactone) tended to increase pAkt with 9 ± 9% (n = 3). This effect was not statistically significant and was not further investigated (Figure 5, panel A). In contrast, there was a clear difference between the effects of ellagic acid and its microbial metabolites. While ellagic acid had a little effect on Akt-phosphorylation (12 ± 4%; n = 3), urolithin A exhibited a significant and reproducible inhibition (35 ± 12%; n = 6; *p* = 0.001 **). Other urolithins (urolithin B, C, D) showed no statistically significant inhibitory effects on Akt-phosphorylation and were not further investigated (n = 1–2, Figure 5, panel B).

### 3.4. Immunoblotting: Effects of Polyphenols on pAkt Ser473 and pAkt Thr308

To confirm results from the ELISA analysis, the short-term effects of eight selected polyphenols from five different structural subclasses (Table 1) were re-examined semi-quantitatively by the Western blot analysis. These compounds were investigated regarding their effects on both phosphorylation sites Ser473 and Thr308 in endothelial EA.hy926 cells.

**Figure 6:**
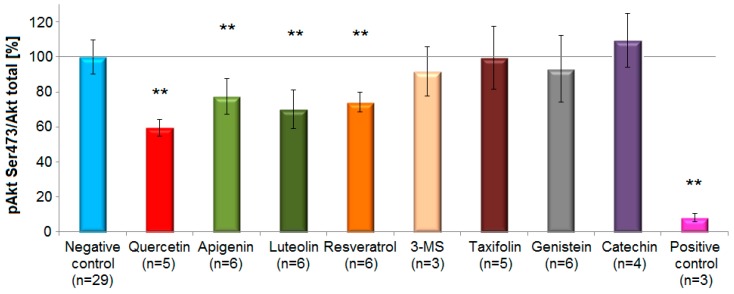
Effects of selected polyphenols on Akt-phosphorylation at Ser473 site in EA.hy926 cells according to the Western blot analysis. Columns represent mean and standard deviation (%). The flavonol quercetin, the flavones luteolin and apigenin, and the stilbenoid resveratrol, caused a statistically significant reduction in Akt-phosphorylation compared to the control (** *p* < 0.01, one-way ANOVA with Tukey HSD test, n = 3–6). On the contrary, 3,4′,5-trimethoxy-trans-stilbene (3-MS), taxifolin, genistein and (+)-catechin revealed no inhibitory potential. Growth factors deprivation overnight served as a positive control.

Representatives from the subclasses flavones, flavonols and stilbenoids caused a statistically significant decrease (*p* < 0.01, one-way ANOVA with Tukey HSD test) of Akt-phosphorylation level after short-term incubation (5 min) compared to controls. The compounds with the most prominent inhibitory effects on pAkt Ser473 (mean inhibition ± standard deviation) were quercetin (40 ± 5%), luteolin (30 ± 11%), resveratrol (26% ± 6%) and apigenin (22.6 ± 10%). In contrast, genistein (7 ± 19%), 3,4′,5-trimethoxy-trans-stilbene (3-MS) (8 ± 14%) caused only a small and statistically non-significant reduction in the phosphorylation of Akt at Ser473 compared to the negative control. Taxifolin (0.6 ± 18%) did not reveal any activity (Figure 6).

As a positive control cells were serum-deprived overnight. Under these conditions the phosphorylation levels at Ser473 and Thr308 strongly decreased by 92 ± 2% (n = 3) and 89 ± 5% (n = 3), respectively. The inhibitory effect of polyphenols on pAkt Thr308 was similar for quercetin (36 ± 3%), luteolin (27 ± 10%), resveratrol (32 ± 13%) and apigenin (28 ± 10%). A minor, not statistically significant increase in Akt-phosphorylation at Ser473 was observed after incubation with (+)-catechin. Likewise, genistein, taxifolin, 3,4′,5-trimethoxy-trans-stilbene and (+)-catechin showed minor and no statistically significant effects on Thr308 (Supplementary Materials, Table S2).

In addition, short-term effects of quercetin on Akt-phosphorylation in primary endothelial cells (HUVEC) were analyzed by Western blot. This model compound reduced the phosphorylation of Akt at Ser473 site by 44 ± 5%. This effect was statistically significant (*p* < 0.01) and very similar to the effects determined in experiments employing the immortalized cell line Ea.hy926 (Supplementary Materials, Table S3).

**Figure 7:**
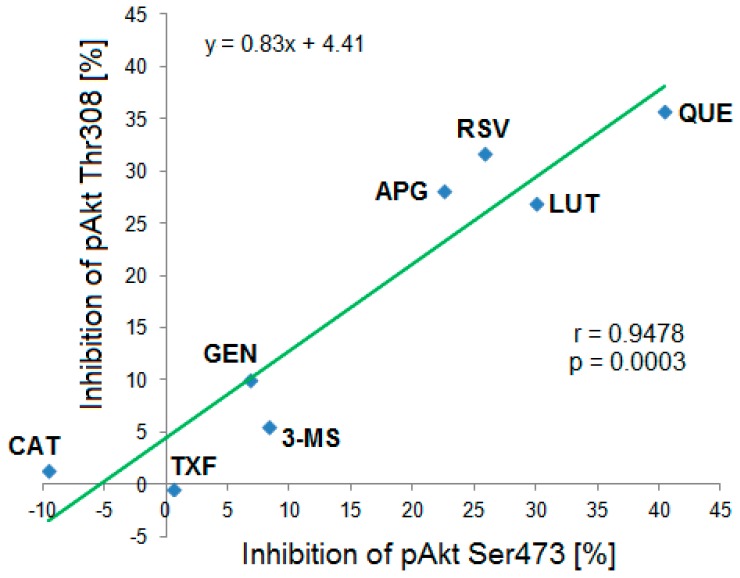
Correlation between the Ser473 and Thr308 phosphorylation status of Akt-kinase after incubation with different polyphenols and analyzed by Western blot. The strong statistically significant correlation (r = 0.9478, *p* = 0.0003, n = 3–6) suggested that the compounds exhibited similar inhibitory effects on both phosphorylation sites. CAT: (+)-catechin, TXF: Taxifolin, GEN: Genistein, 3-MS: 3,4′,5-trimethoxy-trans-stilbene, APG: Apigenin, RSV: Resveratrol, LUT: Luteolin, QUE: Quercetin.

Based on the Western blot results a correlation analysis of mean inhibitory effects of the tested polyphenols on both phosphorylation sites pAkt Ser473 and pAkt Thr308 was performed. The calculated correlation coefficient was r = 0.9478 (R^2^ = 0.898) indicating a strong positive and statistically significant (*p* = 0.0003) correlation between both variables (Figure 7).

## 4. Discussion

The present study quantitatively compared the effects of polyphenols from nine different structural subclasses on pAkt in endothelial cells and identified several active compounds. The consistence of the results from the ELISA and Western blot analysis substantiated their significance and provided a solid basis for structure-activity evaluations and determination of structural key features for pAkt inhibition.

Inhibition of pAkt by quercetin, apigenin, luteolin, and resveratrol was observed in both independent analytical approaches. Likewise, compounds that were determined to lack inhibitory effects in ELISA were only slightly active or inactive in the immunoblotting. Comparison of the quantitative data revealed that the results obtained in both approaches were highly similar for quercetin (immunoblotting compared to ELISA: 40 ± 5% compared to 36 ± 8%) and resveratrol (26 ± 6% compared to 26 ± 5.6%). Bigger differences were observed for luteolin (30 ± 11% compared to 44 ± 18%) and apigenin (22.6 ± 10% compared to 32 ± 6%). However, Western blot analysis is a complex procedure and multiple steps (transfer, stripping, and chemiluminescent detection) can contribute to variations of results. This technique is generally considered as a semi-quantitative method [44]. For this reason, only the data obtained from the screening via ELISA were considered in subsequent analysis of structure-activity relationships.

To the best of our knowledge, the present study is the first comprehensive screening for the effects of polyphenols on pAkt in endothelial cells. The scope of the present study was broad, including representatives from chemical classes such as stilbenoids, urolithins, and phenolic acids, and investigating the effects of methylation and glycosylation. As far as we know, 6-hydroxyflavone, 6-methoxyflavone, 7-methoxyflavone, and pinostilbene have not previously been described as inhibitors of Akt-phosphorylation.

In another study, 24 flavonoids were investigated in adipocytes by Western blot regarding their activities on multiple targets related to insulin-signaling, among which was Akt-kinase [45]. The authors showed that luteolin and kaempferol statistically significantly reduced Akt activity compared to untreated controls. In addition, quercetin, fisetin and apigenin tended to decrease Akt-activity, although their effects were not statistically significant. The authors proposed the insulin receptor and PI3K as direct targets of flavonoids. This is generally in accordance with the outcome of the present investigation showing that some flavonoids are capable to inhibit Akt and that the flavone luteolin displayed high activity.

In a related study employing macrophages the effects of stilbenoids and their semi-synthetic derivatives on the PI3K/Akt-pathway were examined by immunoblotting [46]. Piceatannol, monomethylpinosylvin and pinosylvin were found to be the most potent inhibitors of Akt-phosphorylation. Similarly to the present results, 10 µM resveratrol exhibited clear activity, while its dimethylated derivative pterostilbene was not active at the same concentration.

As Akt is considered as a major downstream effector of PI3K [47], publications investigating the relationships between flavonoid structure and the inhibition of this kinase were also studied. Agullo et al. determined the effects of 14 flavonoids from different subgroups on the enzymatic activities of purified PI3K in vitro [48]. The most active compounds were myricetin, luteolin, apigenin, quercetin, and fisetin. This corresponds well with the data obtained in the present screening. Likewise, flavan-3-ols, flavanones, isoflavones and morin were shown to be inactive. Another study investigating the inhibitory activity of flavonoids against specific class I isoforms of PI3K reported similar results [49]. This might suggest that PI3K is a major molecular target of the investigated polyphenols.

Compared to most previously published papers, both phosphorylation sites of Akt (Ser473 and Thr308) were investigated in the present study, as both sites are important for the full activation of this enzyme [17]. The strong correlation found in the present study suggested that both phosphorylation sites were similarly influenced by polyphenols in the utilized in vitro model. Vincent et al. postulated that pAkt Thr308 is the more important predictor for the Akt-activity and should preferably be analyzed rather than pAkt Ser473 [50].

A bio-activation of ellagic acid was shown for different targets such as heme peroxidases [51] and estrogen receptors [52]. Likewise, the present data suggested a bio-activation of ellagic acid by microbiotic metabolism regarding the diabetes relevant target Akt-kinase in endothelial cells. Among the tested metabolites only urolithin A exhibited a clear inhibition of Akt-phosphorylation (35 ± 12%), in spite of the structural homology to the other urolithins (dibenzo-α-pyrones). This suggests a specific effect of urolithin A. Our results are consistent with a study in bladder cancer cells showing that only urolithin A statistically significantly inhibited the phosphorylation of Akt compared to controls, while urolithin B and C did not cause any changes [53].

Determination of semi-quantitative structure-activity relationships of the polyphenols and their effects on Akt-phosphorylation revealed that the most active compounds belonged to the subclass of flavones, followed by flavonols. For these two groups the structural hallmarks were the double bond between C2 and C3 of the ring C, preferably lack of substitution at the C3 of the ring C, and 3′,4′-catechol group in the ring B. The most active compounds were those with unsubstituted OH-groups (no methylation or glycosylation).

The phosphorylation status of Akt was reported to be dependent on oxidative stress levels [54]. Therefore, the own assumptions on structure-activity relationships regarding Akt-phosphorylation were compared with the antioxidant activity of polyphenols. According to the Bors’ criteria [55] a C2=C3 double bond is beneficial for the antioxidant activity of flavonoids, as it is responsible for the electron delocalization over all tree rings of the system and thus contributes to radical stabilization. In addition, ortho-catechol structure in the ring B is considered important as it assures the stability of flavonoid phenoxyl radical by hydrogen bond. Furthermore, the presence of 3-OH group (ring C) is beneficial for the activity. Akt-phosphorylation inhibition and the antioxidant properties differed from each other as the C-ring OH-group is favorable for radical scavenging activity [43], but negatively influenced the inhibitory potential of polyphenols on pAkt. An additional difference was the effect of glycosylation. It decreased the antioxidant activity compared to the aglycones [56], but seemed to abolish and even reversed the inhibitory activity regarding pAkt in the present study. Therefore, the polyphenol effects on pAkt cannot be solely explained by their antioxidant properties.

Moderate inhibitory effects of polyphenols as observed in the present study might be beneficial in the case of endothelial dysfunction. It has been described that the hyperactive S6K1 (ribosomal protein S6 kinase beta-1) in senescent endothelial cells might contribute to an increased oxidative stress and decreased NO levels. S6K1 is a downstream target of Akt and its over-activation was reported to contribute to insulin resistance [57]. It was shown that resveratrol inhibited Akt/S6K1-signaling and reversed the endothelial dysfunction and hallmarks of aging [58], which is again consistent with the present results.

## 5. Conclusions

The present study for the first time quantitatively compared the influence of polyphenols from nine different subclasses on Akt-phosphorylation in endothelial cells. Quercetin, resveratrol, apigenin and luteolin statistically significantly inhibited the phosphorylation of both Akt Ser473 and Akt Thr308. A differential inhibitory effect on Akt-phosphorylation for urolithin A, but not for other structurally related compounds was uncovered. A semi-quantitative structure-activity analysis suggested functional groups important for the inhibitory activity of polyphenols on Akt-phosphorylation. It was hypothesized that PI3K-inhibition, but not solely the antioxidant properties of those polyphenolic compounds might play a major role for their effects on the Akt-kinase.

## Figures and Tables

**Table 1:** List of polyphenolic compounds and intestinal microbiota-generated metabolites included in the screening regarding their effects on phosphorylation of Akt at Ser473 site. Classification according to Zhang et al. 2016 [2].

Chemical Subclass	Individual Compounds	Number
Flavones *	Luteolin *, apigenin, flavone, 6-hydroxyflavone, 3-hydroxyflavone, 6-methoxyflavone, 7-methoxyflavone, 7,8-dihydroxyflavone, chrysin, baicalein, baicalin, 3-methoxyflavone, 3,4′-dihydroxyflavone, 3-hydroxy-4′-methoxyflavone, vitexin, wogonoside	16
**Flavon-3-ols (Flavonols)**	**quercetin,** fisetin, kaempferol, myricetin, morin	5
**Stilbenoids**	**resveratrol,** pinostilbene, pterostilbene, **3,4′5-trimethoxy-trans-stilbene,** piceatannol	5
**Flavan-3-ols (Flavanols)**	**(+)-catechin, taxifolin,** (−)-epicatechin, (−)-epicatechin gallate, (−)-epigallocatechin gallate, (−)-gallocatechin gallate	6
**Isoflavones**	**genistein**	1
Flavanones	naringenin	1
Phenolic acids	caffeic acid, trans-ferulic acid, chlorogenic acid	3
Catechin metabolites	M1, M2	2
Ellagic acid and its metabolites	ellagic acid, urolithin A, B, C, D	5

* Subclasses and representatives, which were additionally investigated by Western blot analysis, are marked in bold.

**Table 2:** Summary of the semi-quantitative structure-activity relationships of flavones/flavonols and stilbenoids regarding pAkt inhibition.

	Structural Features	Possible Effect	Evidence
**Flavones/Flavon-3-ols**			
1	C2=C3 double bond (Ring C)	Essential	Quercetin/Taxifolin; Apigenin/Naringenin
2	OH-groups (Ring B) (*m*-, *p*-)	Contribution	Luteolin (*m*, *p*) > Apigenin (*p*) > Chrysin (Ø)
3	3-p. (Ring C): hydroxylation	Reduction	Luteolin/Quercetin; Apigenin/Kaempferol
4	Glycosylation	Abolishment	Apigenin/Vitexin; Baicalein/Baicalin
**Stilbenoids**			
1	Three free OH-groups	Optimal	Resveratrol > Pinostilbene > Pterostilbene ≈ 3,4′,5-trimethoxy-trans-stilbene
2	Methylation of OH-groups	Abolishment	

**Table 3:** Comparison of the proposed structure-activity features regarding inhibitory effects on Akt-phosphorylation (pAkt) determined in the present study with the antioxidant properties of polyphenols [43].

Functional Characteristic	Inhibition of pAkt	Antioxidant Activity
Double bond (C2=C3)	Increase	Increase
OH-group in ring A	Increase	Increase
OH-group in ring B	Increase	Increase
**OH-group in ring C (3-OH)** *	**Decrease**	**Increase**
**Glycosyl group** *	**Abolish/Reverse**	**Decrease**
O-Methyl group	Decrease	Decrease

* Functional groups entailing divergent effects are marked in bold and red.

## Supplementary Materials

The following are available online at https://www.mdpi.com/2218-273X/9/6/219/s1, Table S1: Comprehensive screening for effects of polyphenols on Akt-phosphorylation (Ser473) in Ea.hy926 cells: Normalized values, Table S2: Results from Western blot analysis for effects of polyphenols on Akt-phosphorylation (Ser473 and Thr308) in Ea.hy926 cells: Normalized values, Table S3: Effects of quercetin on the phosphorylation status of Akt (Ser 473) in primary endothelial cells (HUVEC): Normalized values.
