# Supplementary material for: Screening of Inhibitory Effects of Polyphenols on Akt-Phosphorylation in Endothelial Cells and Determination of Structure-Activity Features
Source: Biomolecules. 2019 Jun 5;9(6):219. doi: 10.3390/biom9060219 (PMC6627700; doi:10.3390/biom9060219)
Supplement: Supplementary file 1 [file biomolecules-09-00219-s001.pdf]

Supplementary Material S1

Table S1: Comprehensive screening for effects of polyphenols on Akt-phosphorylation (Ser473) in Ea.hy926 cells: normalized values

Group I: Substances with pronounced inhibitory potential (> 20 %)

[%]	Negative control	Quercetin	Resveratrol	Apigenin	Luteolin	6-HF
Mean	100.00	64.29	73.96	68.21	55.69	73.14
Inhibition	0.00	35.71	26.04	31.79	44.31	26.86
S.D.	5.19	8.33	5.58	6.16	17.95	7.78
n	37	6	5	5	6	2

[%]	6-MF	Chrysin	7,8-DHF	Urolithin A	Fisetin	Flavone
Mean	75.11	78.30	79.68	64.72	71.91	77.76
Inhibition	24.89	21.70	20.32	35.28	28.09	22.24
S.D.	10.29	N/A	N/A	11.80	9.09	2.28
n	2	1	1	6	3	2

6-HF= 6-Hydroxyflavone; 6-MF= 6-Methoxyflavone; 7,8-DHF= 7,8-Dihydroxyflavone

Group II: Substances with inhibitory potential between 10 % and 20 %

[%]	Negative control	Pinostilbene	7-MF	Baicalein	Ellagic Acid
Mean	100.00	80.66	81.61	81.82	86.02
Inhibition	0.00	19.34	18.39	18.18	13.98
S.D.	5.19	14.45	N/A	N/A	5.64
n	37	2	1	1	2

[%]	Urolithin D	(-)-GCG	3-MF	3,4'-DHF	Myricetin	Kaempferol
Mean	88.83	87.77	87.15	89.31	81.09	82.98
Inhibition	11.17	12.23	12.85	10.69	18.91	17.02
S.D.	N/A	N/A	N/A	N/A	N/A	11.81
n	1	1	1	1	1	3

7-MF= 7-Methoxyflavone; (-)-GCG= (-)-Gallocatechin gallate; 3-MF= 3-Methoxyflavone; 3,4'-DHF= 3,4'-Dihydroxyflavone

Group III: Substances with slight inhibitory potential between 5 % and 10 %

[%]	Negative control	Genistein	Taxifolin	3-HF	Urolithin B	Morin
Mean	100.00	90.95	91.31	93.66	94.78	90.31
Inhibition	0.00	9.05	8.69	6.34	5.22	9.69
S.D.	5.19	N/A	N/A	4.22	N/A	N/A
n	37	1	1	2	1	1

3-HF= 3-Hydroxyflavone;

Group IV: Substances with no inhibitory potential (Not hits)

[%]	Negative control	Caffeic acid	M1	Urolithin C	EPI	EGCG
Mean	100.00	99.01	100.33	100.08	104.23	102.03
Inhibition	0.00	0.99	-0.33	-0.08	-4.23	-2.03
S.D.	5.19	N/A	N/A	N/A	N/A	N/A
n	37	1	1	1	1	1

[%]	3-H-4'-MF	Wogonoside	Pterostilbene	3-MS	Piacetannol	Chlorogenic Acid
Mean	97.23	105.02	105.96	100.93	102.73	96.41
Inhibition	2.77	-5.02	-5.96	-0.93	-2.73	3.59
S.D.	N/A	N/A	1.59	9.99	N/A	N/A
n	1	1	2	2	1	1

EPI= Epigallocatechin; EGCG= Epigallocatechin gallate; 3-H-4'-MF= 3-Hydroxy-4'-Methoxyflavone; 3-MS= 3,4’,5-Trimethoxy-trans-stilbene;

[%]	(+)-Catechin	(-)-Epicatechin	Naringenin	M2	Ferulic acid	Baicalin	Vitexin
Mean	105.10	110,73	107,32	109.35	108.32	110.85	108,51
Inhibition	-5.10	-10,73	-7,32	-9.35	-8.32	-10.85	-8,51
S.D.	17.14	4.90	N/A	8.90	21.35	N/A	N/A
n	4	3	1	3	3	1	1

Table S2: Results from Western blot analysis for effects of polyphenols on Akt-phosphorylation (Ser473 and Thr308) in Ea.hy926 cells: normalized values

A) pAkt Ser473

[%]	Negative control	Quercetin	Resveratrol	Apigenin	Luteolin
Mean	100.00	59.54	74.15	77.43	70.04
Inhibition	0.00	40.46	25.85	22.57	29.96
S.D.	9.73	4.83	5.72	10.30	11.06
n	29	5	6	6	6

[%]	(+)-Catechin	Genistein	Taxifolin	3-MS	Positive control
Mean	109.51	93.23	99.44	91.68	8.16
Inhibition	-9.51	6.67	0.56	8.32	91.84
S.D.	15.35	18.98	17.96	14.16	2.18
n	4	6	5	3	3

3-MS= 3,4’,5-Trimethoxy-trans-stilbene; Positive control – serum free starving overnight

B) pAkt Thr308

[%]	Negative control	Quercetin	Resveratrol	Apigenin	Luteolin
Mean	100.00	64.29	68.22	71.90	73.10
Inhibition	0.00	35.71	31.78	28.10	26.90
S.D.	9.08	3.73	13.09	10.21	10.33
n	24	3	4	6	6

[%]	(+)-Catechin	Genistein	Taxifolin	3-MS	Positive control
Mean	98.64	89.95	100.31	94.42	11.36
Inhibition	1.36	10.05	-0.31	5.58	88.64
S.D.	10.51	14.59	10.21	21.41	5.33
n	4	4	5	3	3

3-MS= 3,4’,5-Trimethoxy-trans-stilbene; Positive control – serum free starving overnight

Table S3: Effects of quercetin (10 µM, 5 min) on the phosphorylation status of Akt (Ser473) in primary endothelial cells (HUVEC): normalized values

[%]	Negative control	Quercetin
Mean	100.00	56.29
Inhibition	0.00	43.71
S.D.	9.51	4.97
n	5	5

p= 0.0007

Negative control [%]	Quercetin [%]
100.00	62.01
103.82	63.49
100.00	61.12
123.06	59.71
100.00	50.28
